# Supplementary material for: Transcriptome Analysis of White- and Red-Fleshed Apple Fruits Uncovered Novel Genes Related to the Regulation of Anthocyanin Biosynthesis
Source: Int J Mol Sci. 2024 Feb 1;25(3):1778. doi: 10.3390/ijms25031778 (PMC10855924; doi:10.3390/ijms25031778)
Supplement: Supplementary file 1 [file ijms-25-01778-s001.zip › Supplementary tables and figures captions.pdf]

Supplementary Table S1. The effective data volume and quality of sequence reads**.**

Supplementary Table S2. Gene IDs and GO enrichment.

Supplementary Table S3. RNAseq filtered data of gene expression profiles and gene annotation.

Supplementary Table S4. Differentially Expressed Genes and GO term categorization**.**

Supplementary Table S5. Categories of the first and the second round of gene classification.

Supplementary Figure S1. Groups of gene ontology classification.

Supplementary Figure S2. Top GO terms extracted from the data.

Supplementary Figure S3. Genes classified in top GO terms of the Biological Process category.

Supplementary Figure S4. Genes classified in top GO terms of the Cellular Components category.

Supplementary Figure S5. Genes classified in top GO terms of the Molecular Function category.

Supplementary Figure S6. Red Love 'General' vs. 'Early Fuji' comparison of up- and down-regulation of genes classified in KEGG pathways.
