# Supplementary figures and images for: Transcriptome Analysis of White- and Red-Fleshed Apple Fruits Uncovered Novel Genes Related to the Regulation of Anthocyanin Biosynthesis
Source: Int J Mol Sci. 2024 Feb 1;25(3):1778. doi: 10.3390/ijms25031778 (PMC10855924; doi:10.3390/ijms25031778)

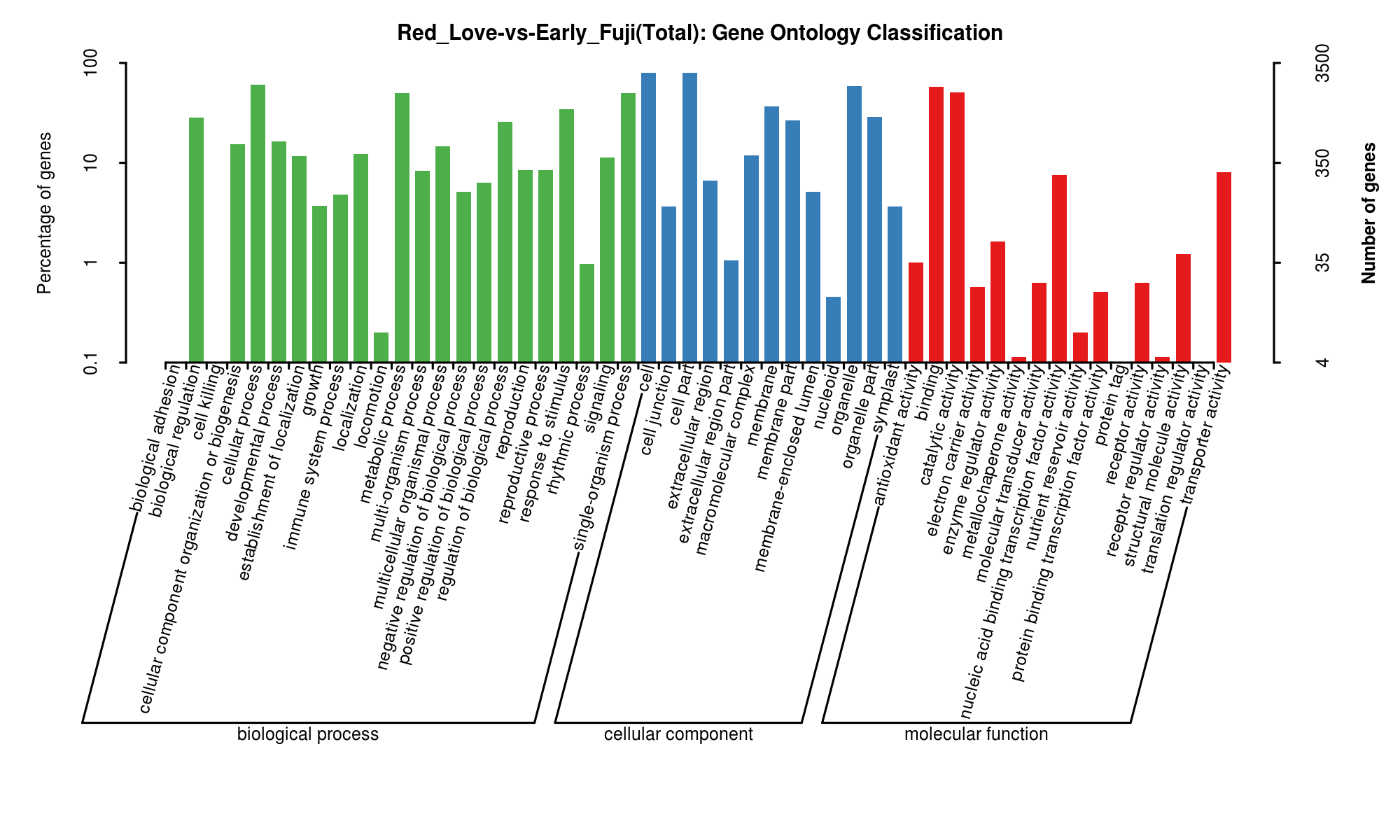

Supplement: Supplementary file 1 [file ijms-25-01778-s001.zip › Supplementary Figure S1.png]

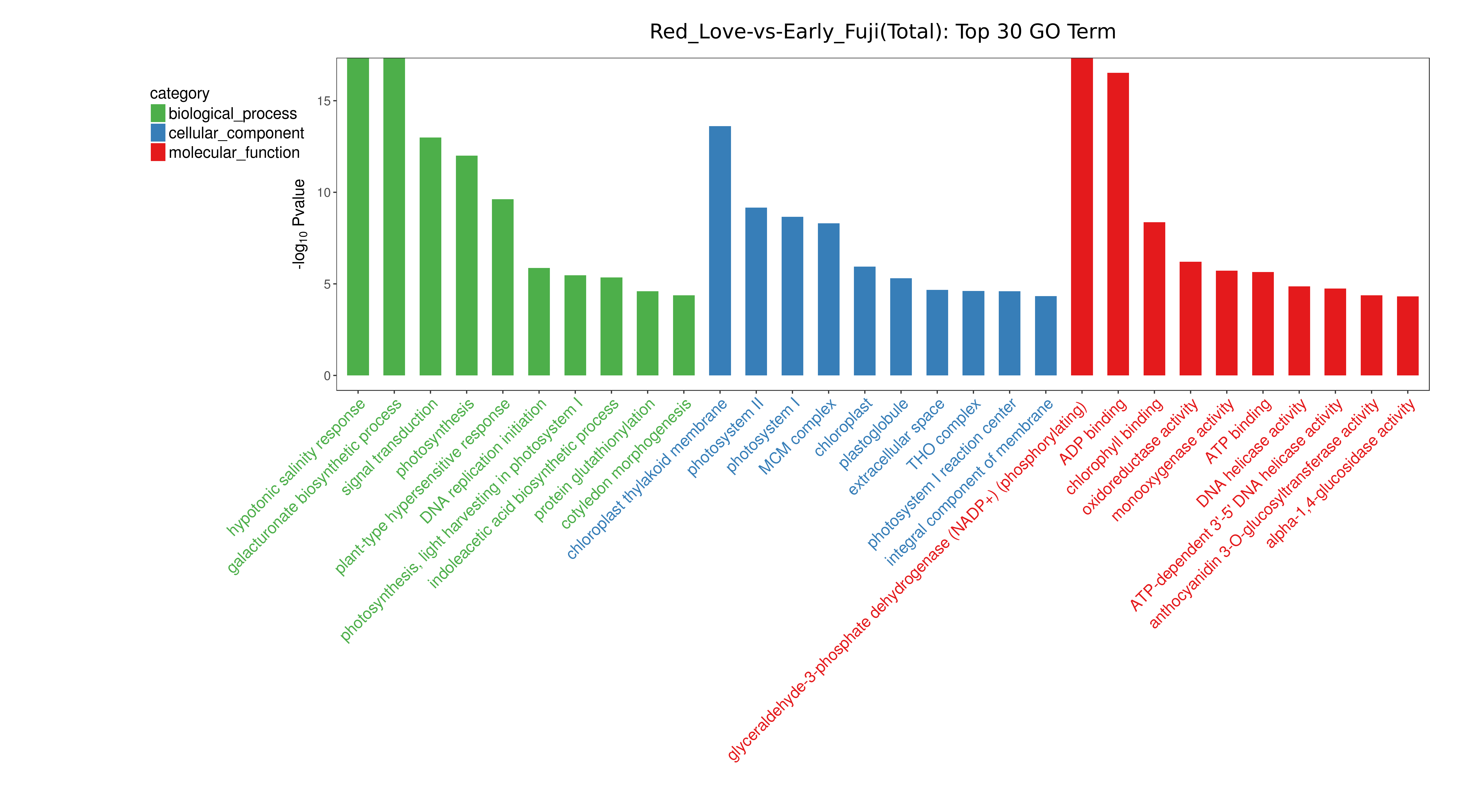

Supplement: Supplementary file 1 [file ijms-25-01778-s001.zip › Supplementary Figure S2.png]

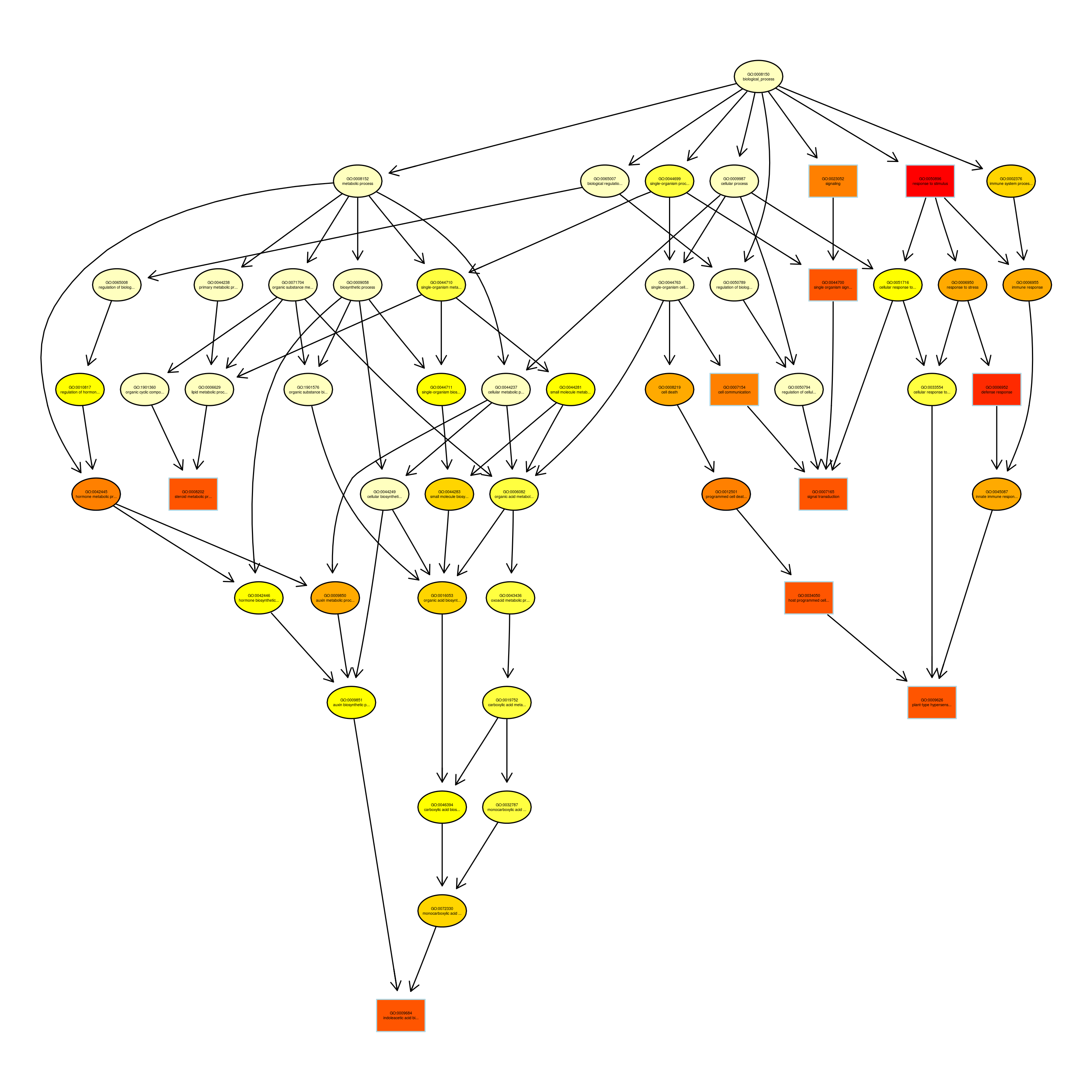

Supplement: Supplementary file 1 [file ijms-25-01778-s001.zip › Supplementary Figure S3.png]

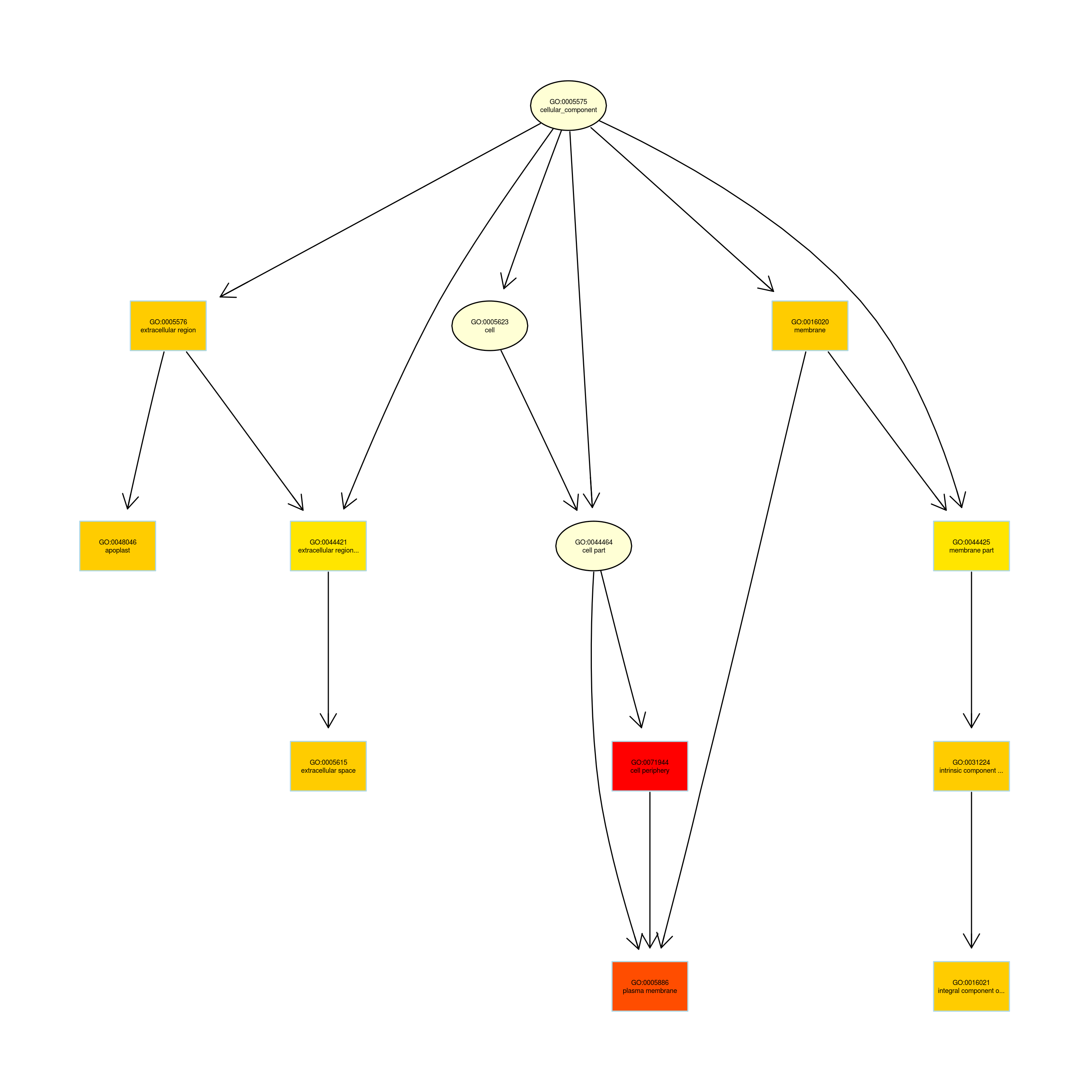

Supplement: Supplementary file 1 [file ijms-25-01778-s001.zip › Supplementary Figure S4.png]

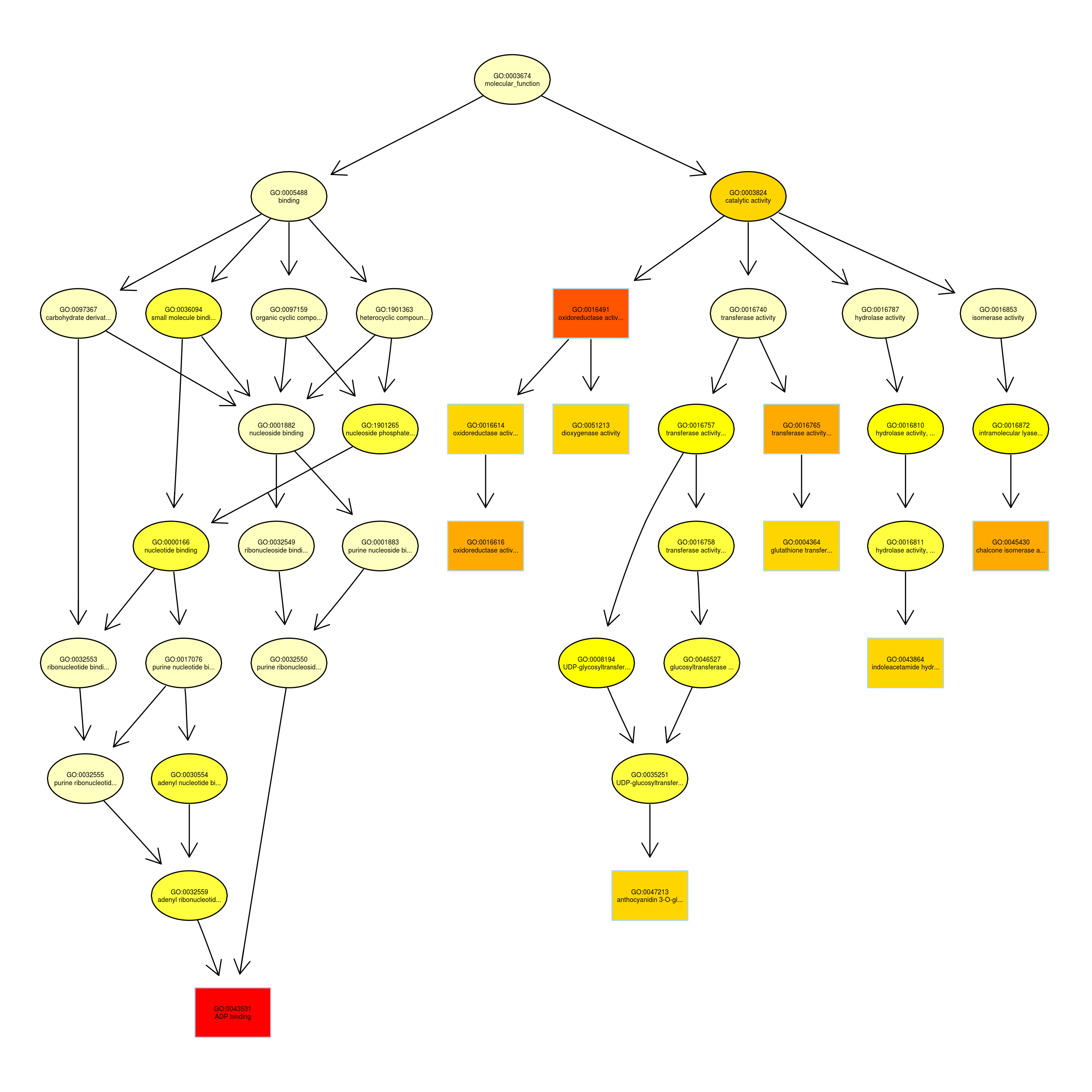

Supplement: Supplementary file 1 [file ijms-25-01778-s001.zip › Supplementary Figure S5.png]

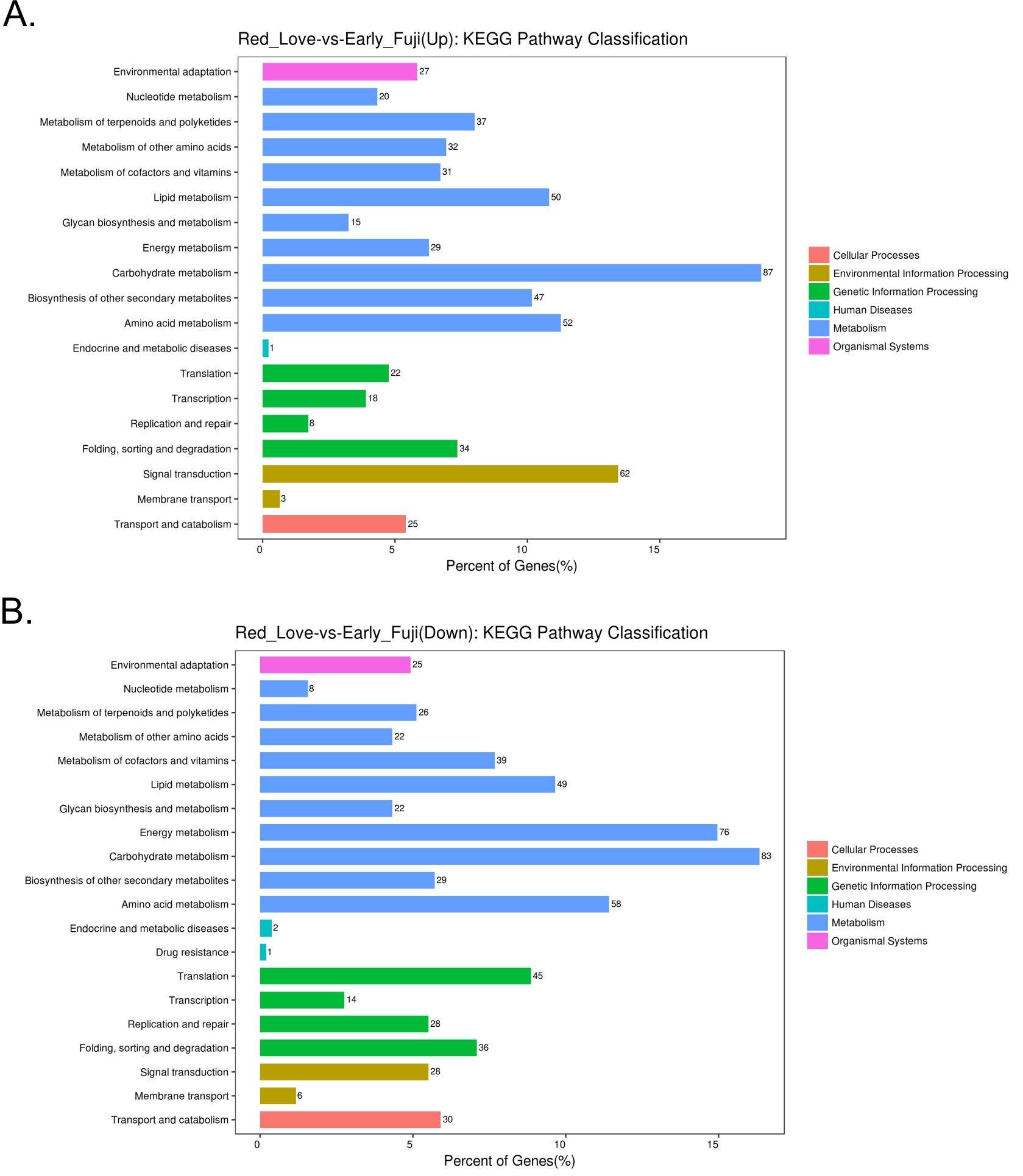

Supplement: Supplementary file 1 [file ijms-25-01778-s001.zip › Supplementary Figure S6.jpg]
